# Quantification of Methylene Blue and Evaluation of Its Pharmacokinetics in ICR Mice by Liquid Chromatography‐Quadrupole Time‐of‐Flight Mass Spectrometry Using Difluoroacetic Acid

**DOI:** 10.1002/bmc.70080

**Published:** 2025-04-20

**Authors:** Seo‐jin Park, Juwon Lee, Sangsoo Hwang, Jeong‐hyeon Lim, Hyunjin Cho, Young G. Shin

**Affiliations:** ^1^ Institute of Drug Research and Development, College of Pharmacy Chungnam National University Daejeon South Korea

**Keywords:** brain penetration, difluoroacetic acid, LC‐qTOF‐MS, methylene blue, pharmacokinetics

## Abstract

Methylene blue (MB), a phenothiazine derivative, is currently under clinical trials for Alzheimer's disease (ad) due to its potential to inhibit tau aggregation, a key pathological process in ad. In this study, we developed and qualified a rapid and reliable liquid chromatography‐quadrupole time‐of‐flight mass spectrometry (LC‐qTOF‐MS) method for the quantification of MB in mouse plasma and brain samples. Chromatographic separation was achieved using a PolymerX RP‐1 100 Å (50 × 2 mm, 5 μm) column with a mobile phase consisting of water and methanol containing 0.5% difluoroacetic acid, delivered at a flow rate of 0.5 mL/min. Calibration curves were constructed using quadratic regression (weighted 1/concentration^2^) over a range of 3.05–2222.22 ng/mL in both matrices. The method was successfully applied to characterize the pharmacokinetics of MB in male ICR mice, revealing a high systemic clearance (65.64 mL/min/kg) and substantial brain penetration, as indicated by a brain‐to‐plasma partition coefficient (*K*_*p,brain*_) of 23.50 following single intravenous bolus administration. These findings provide critical insights into MB's in vivo behavior and demonstrate the utility of this bioanalytical method for evaluating MB in preclinical studies.

## 1 Introduction

Alzheimer's disease (ad) is the most common type of dementia, accounting for 60%–80% of all dementia cases (Erkkinen et al. 2018). As of 2024, an estimated 6.93 million people in the United States are living with ad, and this number is projected to rise to 13.85 million by 2060 (Rajan et al. 2021).

The etiology of ad is complex and multifactorial. Key pathological features include the accumulation of amyloid‐beta plaques and abnormal tau proteins, along with acetylcholine deficiency, neuroinflammation, oxidative stress, metal ion imbalance, glutamate dysregulation, insulin resistance, gut microbiota alterations, disrupted cholesterol homeostasis, mitochondrial dysfunction, and impaired autophagy (Zhang et al. 2024). Among these, the formation of amyloid‐beta plaques and tau neurofibrillary tangles (NFTs) is a defining pathological feature of ad (DeTure and Dickson 2019). The accumulation of these pathological lesions disrupts neurotransmitter balance and induces neuronal death, ultimately leads to cognitive impairment (Yiannopoulou and Papageorgiou 2013). Consequently, therapeutic strategies focused on restoring neurotransmitter homeostasis and preventing or eliminating abnormal protein aggregates remain central to ad treatment efforts.

FDA‐approved drug therapies for ad fall into two categories: (1) symptomatic treatments that address neurotransmitter imbalances, including cholinesterase inhibitors (donepezil, rivastigmine, and galantamine) and the N‐methyl‐D‐aspartate receptor antagonist (memantine); and (2) disease‐modifying therapies targeting the underlying pathology, such as anti‐amyloid‐beta antibody therapies (aducanumab, lecanemab, and donanemab), which bind to amyloid‐beta and promote plaque clearance by microglia (Cummings et al. 2024). While neurotransmitter‐modulating drugs alleviate symptoms without affecting disease progression, anti‐amyloid‐beta therapies are limited by adverse effects and modest clinical efficacy. These limitations highlight the need for alternative therapeutic approaches. To address this, recent research has increasingly focused on novel therapeutic strategies that target not only amyloid‐beta but also tau proteins (Jeremic et al. 2023).

Tau protein plays a crucial role in microtubule assembly and stabilization in normal neurons (Iqbal et al. 2010). However, in ad patients, tau undergoes various posttranslational modifications, including hyper‐phosphorylation, acetylation, N‐glycosylation, and truncation, leading to structural misfolding and the formation of aggregates such as tau oligomers, granular tau oligomers, and NFTs. The accumulation of these abnormal tau species contributes to neuronal death (Congdon and Sigurdsson 2018; Huang, Neurochemistry Laboratory, and Harvard Medical School 2020; Soeda and Takashima 2020). Studies suggest that tau oligomers, the precursors to tau filaments, may exhibit greater toxicity in ad patients (Akihiko Takashima 2020; Niewiadomska et al. 2021; Shafiei et al. 2017). Consequently, developing drugs that inhibit tau aggregation or promote its clearance has emerged as a key therapeutic strategy. Among the various tau‐targeting treatments under development, MB stands out as a promising tau aggregation inhibitor (Peng et al. 2023; Soeda and Takashima 2020).

To the best of the authors' knowledge, despite growing interest in MB for its tau‐inhibiting effects, no analytical method has been reported for its quantification in the brain. Most existing bioanalytical methods focus solely on MB quantification in plasma or whole blood, leaving a critical gap in understanding its distribution in the central nervous system (Burhenne et al. 2008; Kim et al. 2014; Rengelshausen et al. 2004; Yang et al. 2011). In contrast, this study presents a comprehensive bioanalytical method that enables MB quantification not only in plasma but also in brain homogenate. Given MB's potential as a tau aggregation inhibitor, the ability to accurately measure its concentration in the brain is crucial for advancing research on its therapeutic applications inneurodegenerative diseases such as ad.

Furthermore, while previous studies have employed various separation techniques for MB quantification, this study is the first to utilize difluoroacetic acid (DFA) as a mobile phase additive in reversed‐phase liquid chromatography‐mass spectrometry (RPLC–MS). DFA has been increasingly recognized for its ability to enhance chromatographic performance in reversed‐phase liquid chromatography (RPLC) while minimizing the loss of mass spectrometry sensitivity (Fu et al. 2023; Lardeux et al. 2021; Nguyen et al. 2019). Its incorporation effectively mitigates peak broadening and long run times, common challenges in previous studies, thereby enabling the development of a high‐throughput bioanalytical method with improved resolution and efficiency.

The optimized method was successfully applied to pharmacokinetic study samples, enabling a comprehensive characterization of MB's pharmacokinetics in ICR mice, including both plasma pharmacokinetics and brain distribution. Moreover, this bioanalytical method is expected to be applicable to MB derivatives and other structurally similar compounds, expanding its potential in bioanalytical research.

## 2 Materials and Methods

### 2.1 Chemical and Reagents

MB, verapamil (used as an internal standard, ISTD) and DFA were purchased from Sigma‐Aldrich (St. Louis, MO, USA). Liquid chromatography‐grade methanol (MeOH) and distilled water (DW) for sample preparation were obtained from SAMCHUN (Seoul, South Korea), while dimethyl sulfoxide (DMSO) was purchased from DAEJUNG (Siheung, South Korea). Mass spectrometry‐grade formic acid (FA), MeOH, and DW were purchased from Thermo Fisher (Massachusetts, USA).

Pooled male ICR mouse plasma in sodium heparin was obtained from Biomedex (Seoul, South Korea). All other reagents were commercially available for bioanalytical use and were used without further purification.

### 2.2 Equipment and Liquid Chromatography‐Quadrupole Time‐of‐Flight Mass Spectrometry (LC‐qTOF‐MS) Conditions

The analytical system comprised a CTC HTS PAL autosampler (LEAP Technologies, Carrboro, NC, USA), two Shimadzu Prominence LC‐20ad pumps, a Shimadzu CBM‐20A HPLC pump controller (Shimadzu, Columbia, MD, USA), and a TripleTOF 5600 quadrupole time‐of‐flight mass spectrometer equipped with a Duospray ion source (Sciex, Foster City, CA, USA).

For LC separation, the PolymerX RP‐1 100 Å (50 × 2 mm, 5 μm) column (Phenomenex, Torrance, CA, USA) was used for the separation of MB and its ISTD, verapamil. The injection volume was 7 μL, with the column temperature maintained at 55°C. The mobile phase flow rate was 0.5 mL/min. Mobile phase A consisted of 0.5% (v/v) DFA in DW, while mobile phase B contained 0.5% (v/v) DFA in MeOH. The gradient conditions are detailed in Table 1.

**TABLE 1:** Mobile phase B (%) in LC gradient elution.

Time (min)	Mobile phase B (%)
0	30
0.2	30
1.0	100
2.0	100
2.3	30
3.5	30

For mass spectrometry, electrospray ionization in positive mode was used for analyte ionization. Ion source gas 1 and 2 were set at 50 psi, and the curtain gas flow rate was 33 L/min. The ion source temperature was maintained at 500°C, and the ion spray voltage was 5500 V. The mass‐to‐charge (m/z) transitions were as follows: m/z 284.1–268.1 for MB and m/z 455.3–165.1 for verapamil. The declustering potential was set at 100 V for MB and 125 V for verapamil, while the collision energy was 30 V for both compounds.

Data acquisition was performed using Analyst TF software (version 1.6), and data processing was conducted with MultiQuant software (version 3.0.3) (Sciex, Foster City, CA, USA).

### 2.3 Preparation of Stocks, Standard (STD), and Quality Control (QC) Samples

A 1 mg/mL MB stock solution was prepared in DMSO, along with a 0.1 mg/mL sub‐stock solution, also in DMSO. Both solutions were stored at −20°C until use.

For the preparation of STD and QC samples, the 0.1 mg/mL sub‐stock solution was serially diluted in DMSO to prepare working solutions. Each working solution was then spiked into blank mouse plasma and blank mouse brain homogenate, followed by the addition of MeOH containing 100 ng/mL of ISTD (verapamil) and 0.1% (v/v) DFA. The final concentrations of the STD samples were 3.05, 9.15, 27.44, 82.31, 246.91, 740.74, and 2222.22 ng/mL in both matrices. The final concentrations of QC samples were 15.03, 165.29, and 1818.18 ng/mL in both matrices.

The in‐house acceptance criteria for accuracy (relative error, RE, %) and precision (relative standard deviation, RSD, %) of the STD and QC samples were set within ±25%, except for the lower limit of quantification (LLOQ) sample, which was within ±30%. These criteria, commonly applied during the drug discovery stage, were adhered to in all quantification and qualification experiments conducted in this study (Bateman et al. 2013; Xu et al. 2005).

### 2.4 Preparation of Samples

#### 2.4.1 Processing of Mouse Plasma Samples

A 20 μL mouse plasma sample was transferred into a 1.5 mL Eppendorf tube and mixed with 100 μL of MeOH containing 100 ng/mL of ISTD (verapamil) and 0.1% (v/v) DFA. The mixture was vortexed and centrifuged at 15,871 rcf for 5 min at 4°C. The resulting supernatant was diluted 1:1 with DW, and a 7 μL aliquot was injected for analysis.

#### 2.4.2 Processing of Mouse Brain Samples

The mouse brain was weighed, and 1× phosphate‐buffered saline (PBS) was added at a volume twice the brain weight, followed by homogenization (resulting in a total threefold dilution). A 30 μL aliquot of the brain homogenate was transferred to a 1.5 mL Eppendorf tube and mixed with 150 μL of MeOH containing 100 ng/mL of ISTD (verapamil) and 0.1% (v/v) DFA. The mixture was vortexed and centrifuged at 15,871 rcf for 5 min at 4°C. The resulting supernatant was diluted 1:1 with DW, and a 7 μL aliquot was injected for analysis.

### 2.5 Qualification of the Bioanalytical Method

To evaluate the robustness and reproducibility of the developed bioanalytical method, linearity, intra‐ and inter‐day accuracy and precision, as well as stability tests, were conducted. Qualification parameters were selected based on a fit‐for‐purpose approach.

#### 2.5.1 Linearity, Selectivity, and Sensitivity of MB

STD samples at seven different concentrations and QC samples at three different concentrations were prepared and analyzed. Calibration curves were constructed by plotting the STD sample concentration as the independent variable (x) and the peak area ratio of the analyte to the ISTD as the dependent variable (y), using quadratic regression (y = ax^2^ + bx + c) with a weighting factor of 1/x^2^. Linearity was assessed based on the coefficient of correlation (*r*), which should be > 0.99.

Selectivity and sensitivity were evaluated using chromatographic peaks of the LLOQ sample and blank samples with or without ISTD (BI or BB) in mouse plasma and brain homogenate.

#### 2.5.2 Intra‐ and Inter‐Day Accuracy and Precision Test

To confirm the robustness and reproducibility of the developed bioanalytical method for quantifying MB in mouse plasma and brain homogenate, fresh STD and QC samples were prepared and analyzed over three separate days. The QC concentrations were set at low (15.03 ng/mL), medium (165.29 ng/mL), and high (1818.18 ng/mL).

#### 2.5.3 Short‐Term (Bench‐Top), Long‐Term, and Freeze–Thaw Stability Tests

The stability of MB in mouse blank plasma and mouse brain homogenate was evaluated under the short‐term (bench‐top), long‐term, and freeze–thaw conditions. QC samples at three concentration levels were subjected to the following conditions; Short‐term stability: incubation at room temperature (RT) for 4 h. Long‐term stability: storage at −80°C for 2 weeks. Freeze–thaw stability: three freeze–thaw cycles between −80°C and RT. Following each storage condition, QC samples were quantified alongside freshly prepared STD samples. Stability was confirmed if the accuracy and precision of QC samples remained within ±25% of the acceptance criteria.

### 2.6 Pharmacokinetic Study in Male ICR Mouse

Male ICR mice (7‐weeks old, 30–33 g) were purchased from SAMTAKO (Osan, South Korea) and used for plasma and brain pharmacokinetic studies. All animal experiments were conducted in accordance with the “Guidelines for the Use of Animals” established by the Chungnam National University Institutional Animal Care and Use Committee and were approved under protocol No. 202404A‐CNU‐071.

#### 2.6.1 Plasma Pharmacokinetic Study

MB was dissolved in 100% normal saline and administered at a dose of 2 mg/kg via a single intravenous (IV) bolus injection through the tail vein. Blood samples were collected from the retro‐orbital plexus at 2‐, 5‐, 15‐, 30‐, 60‐, 120‐, 240‐, 480‐, and 1440‐min postdose using heparinized capillary tubes. The collected blood was centrifuged at 15,871 rcf for 5 min at 4°C, and the resulting plasma supernatant was transferred to a new 1.5 mL Eppendorf tube. Plasma samples were stored at −80°C until analysis.

#### 2.6.2 Brain Distribution Study

To assess the extent of MB brain distribution, brain and plasma samples were collected at 15, 30, 60 and 120 min following single IV bolus administration at 2 mg/kg. At each designated time point, mice (*n* = 3 per time point) were anesthetized with isoflurane and perfused with 20 mL of 1× PBS via cardiac puncture. Brain and plasma samples were then stored at −80°C until analysis.

The *K*_*p,brain*_ value was calculated using the following equation (Gupta et al. 2006).

(1) $$K_{p,\text{brain}}=\frac{{\mathrm{AUC}}_{\text{brain},15-120\;\min}}{{\mathrm{AUC}}_{\text{plasma},15-120\;\min}}$$

where *AUC*_*brain,*15–120 *min*_ and *AUC*_*plasma,*15–120 *min*_ represent the area under the curve of concentration versus time in the brain and plasma, respectively, from 15‐ to 120‐min postdose.

#### 2.6.3 Calculation of Pharmacokinetic Parameters

Pharmacokinetic parameters were calculated using noncompartmental analysis (NCA) in Phoenix WinNonlin software (version 8.1; Pharsight Corporation, Mountain View, CA, USA).

## 3 Results

### 3.1 Method Development

#### 3.1.1 MS/MS spectrum of MB

**FIGURE 1:**
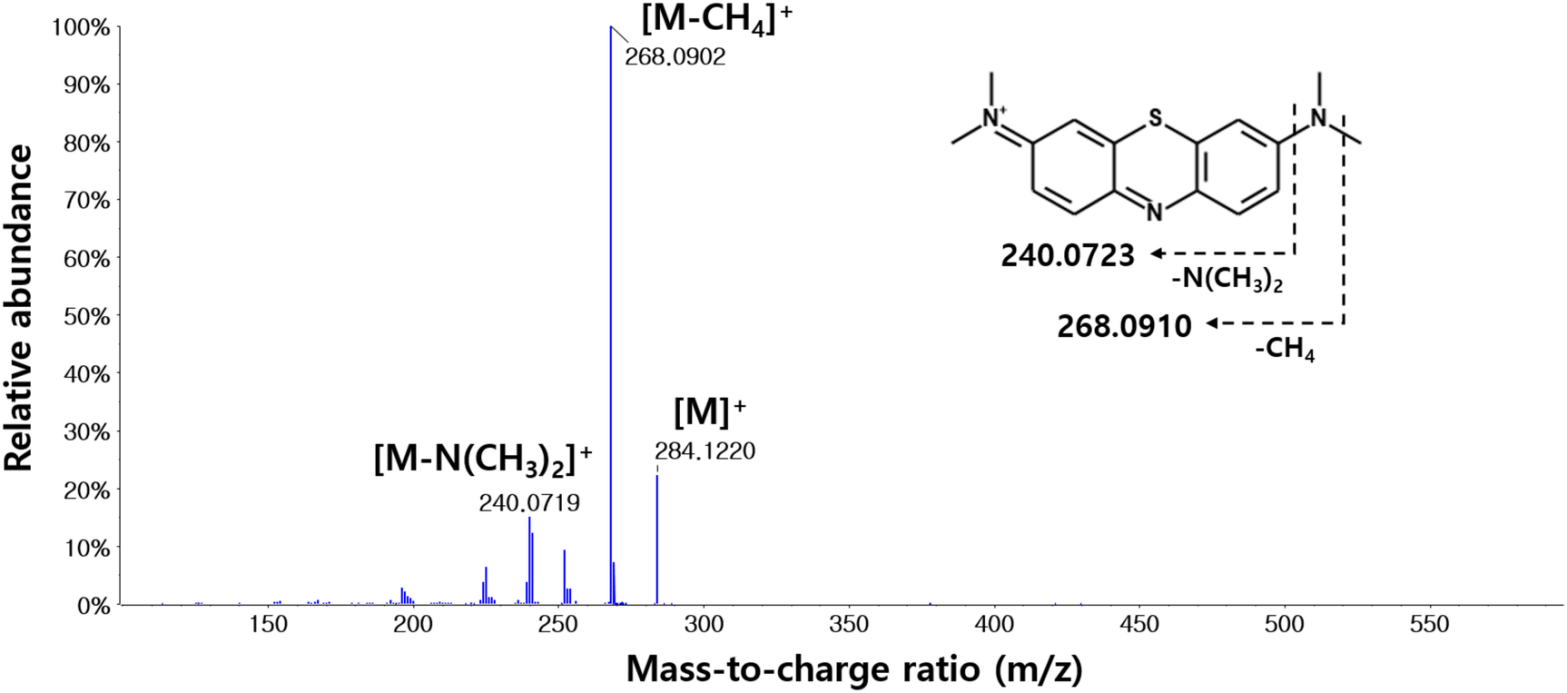
MS/MS spectrum of MB with observed mass‐to‐charge ratio and its predicted fragment ions with theoretical mass‐to‐charge ratio.

The parent ion of MB was detected at *m/z* 284.1, with the most abundant product ion at *m/z* 268.1. The MS/MS spectrum of parent ion and its fragment ions are shown in Figure 1.

#### 3.1.2 Optimization of LC Conditions for the Separation of MB

**FIGURE 2:**
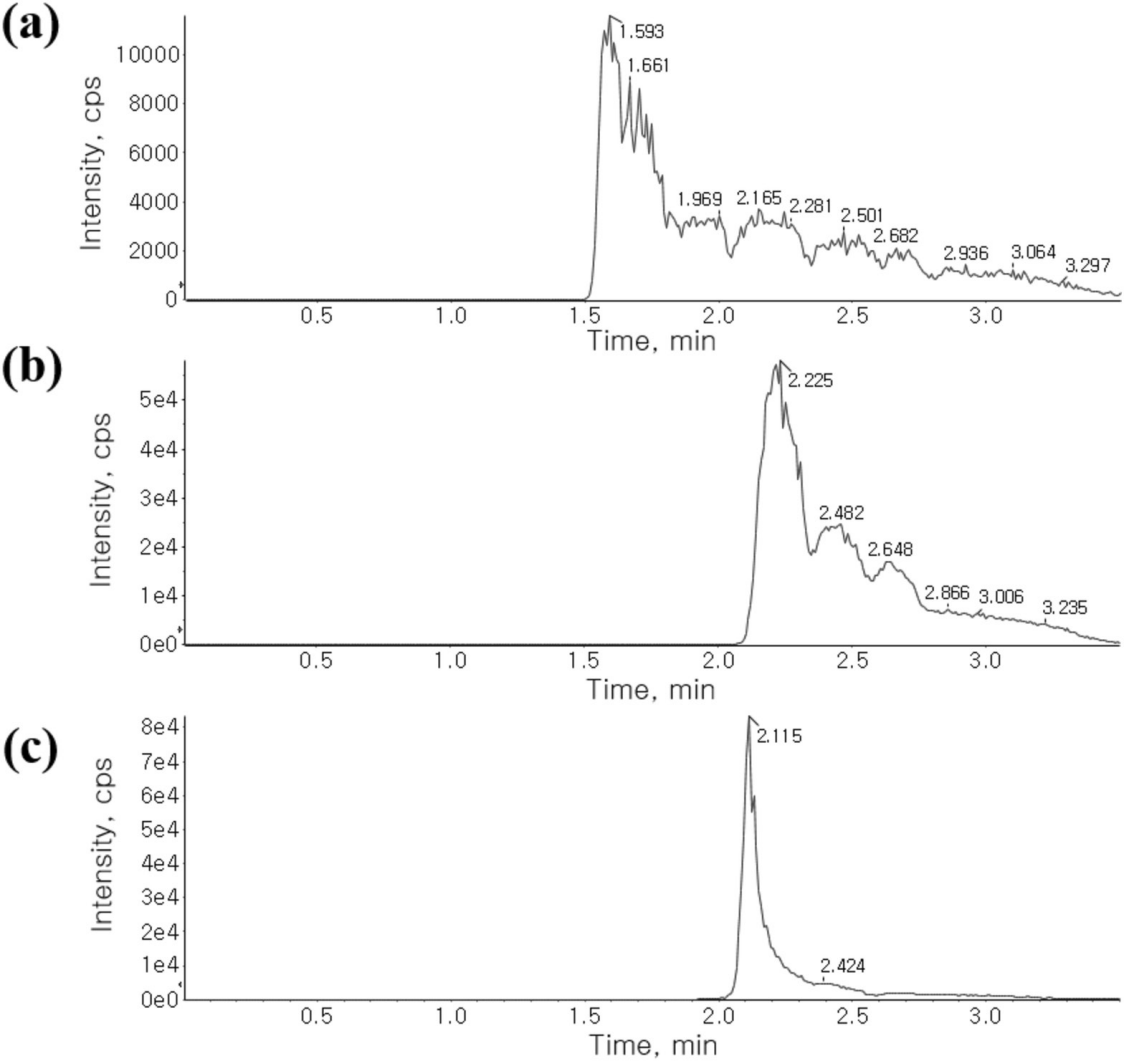
Extracted ion chromatograms of MB (m/z 284.1 to 268.1) under various conditions following protein precipitation from mouse plasma spiked with MB (1818.18 ng/mL).

When analyzed using a Phenomenex Kinetex XB‐C18 column (50 × 2.1 mm, 2.6 μm) with 0.1% (v/v) FA in DW/MeOH, poor chromatographic retention was observed (Figure 2a). Replacing the Kinetex XB‐C18 column with a PolymerX RP‐1 100 Å column (50 × 2 mm, 5 μm) slightly improved peak shape, but chromatographic retention remained inadequate (Figure 2b).

To enhance the interaction between MB and the stationary phase, the mobile phase additive was changed from FA to DFA, leading to a significant improvement in chromatographic performance (Figure 2c).

**FIGURE 3:**
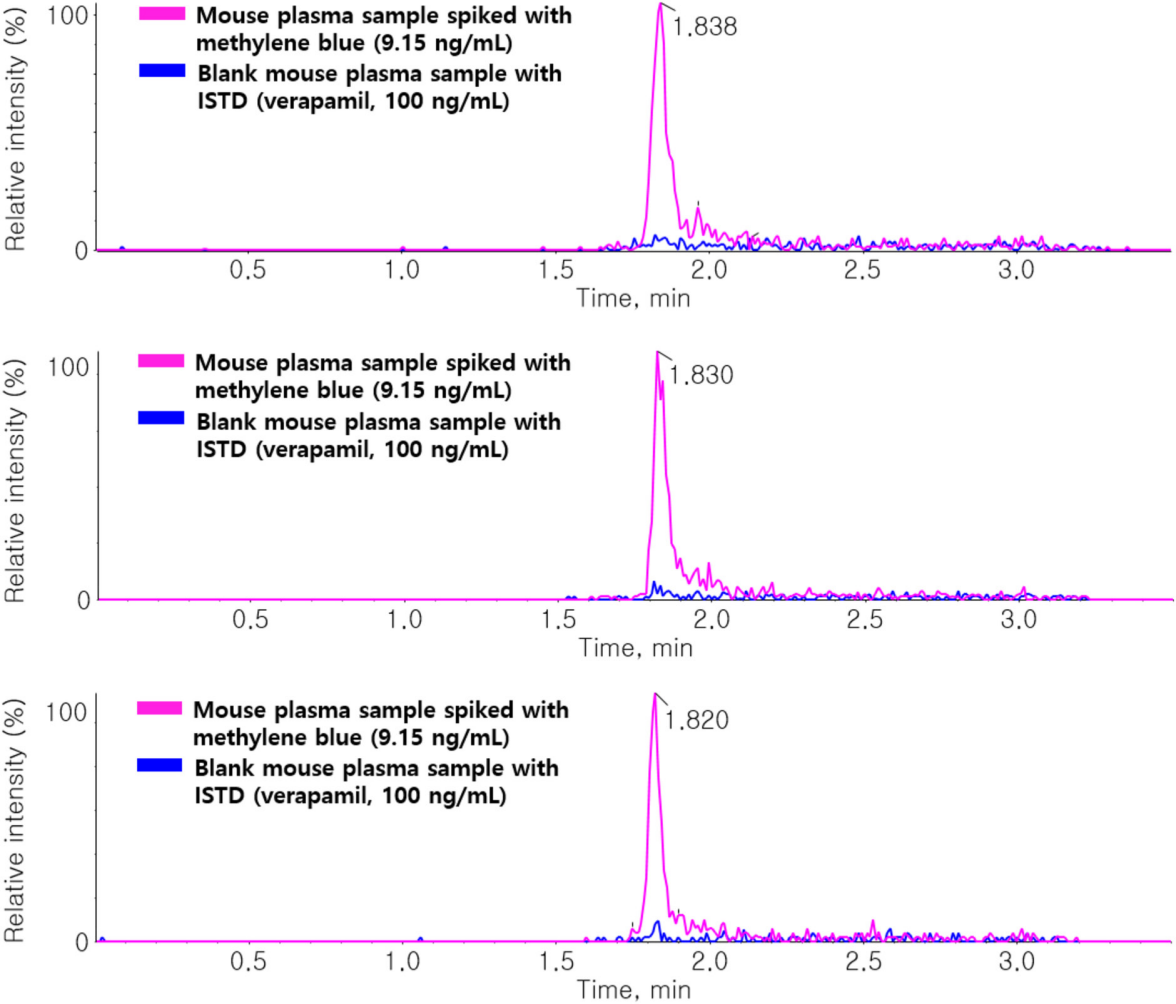
Chromatograms of MB spiked into mouse blank plasma at concentrations of 9.15 ng/mL, using different concentrations of DFA as mobile phase additive in DW and MeOH (mobile phase flow rate: 0.5 mL/min). Panels (a) 0.1% DFA, (b) 0.25% DFA, and (c) 0.5% DFA.

Despite these adjustments, MB peak tailing persisted. To address this issue, the mobile phase flow rate was increased from 0.4 to 0.5 mL/min. Additionally, the DFA concentration was optimized at 0.1%, 0.25%, and 0.5% to refine peak shape. The differences in peak shape at various concentrations of DFA were subtle; 0.5% DFA produced the sharpest and narrowest peaks (Figure 3). Consequently, 0.5% DFA was selected for the final experiments. The PolymerX RP‐1 100 Å, capable of withstanding a pH range from 0 to 14, was chosen.

### 3.2 Qualified Method for MB in Mouse Blank Plasma and Brain Homogenate

#### 3.2.1 Linearity, Selectivity, and Sensitivity of MB

**FIGURE 4:**
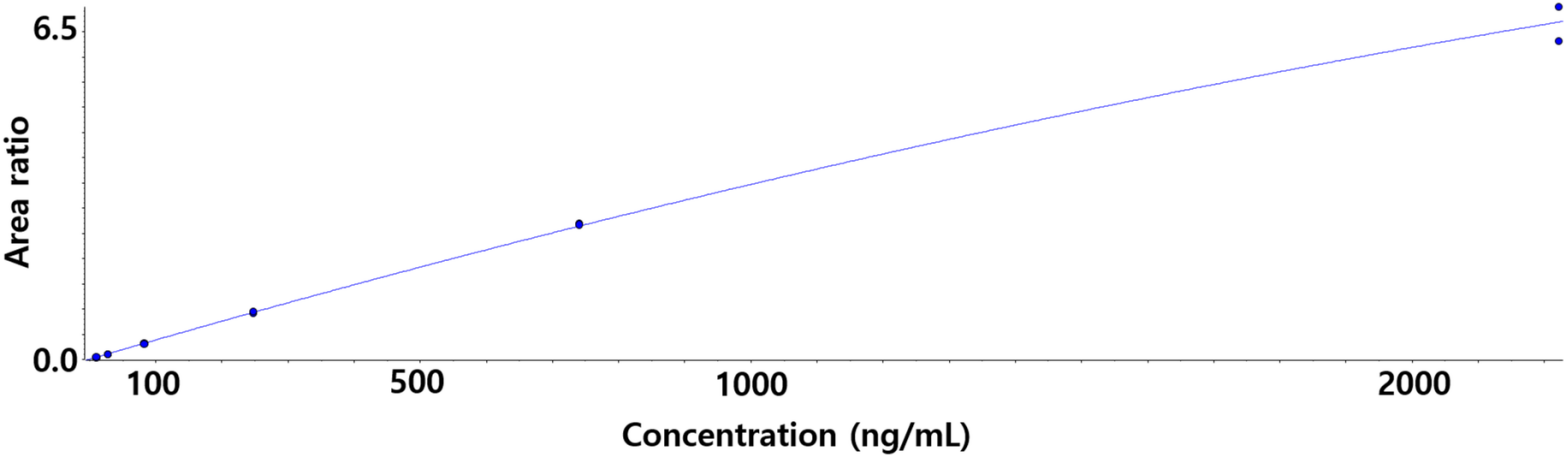
Representative calibration curve for the quantification of MB in blank mouse plasma (range = 3.05–2222.22 ng/mL and *r* value = 0.99532). The curve range and *r* value in blank mouse brain homogenate were 3.05–2222.22 ng/mL and 0.99643, respectively (data not shown).

The calibration curve range was 3.05–2222.22 ng/mL in blank plasma and brain homogenate. The representative regression equations were y = −1.59284e − 7x^2^ + 0.00341x + 0.01104 (weighting factor: 1/x^2^, *r* = 0.99532) in blank plasma and y = −6.00019e − 7x^2^ + 0.00314x + 0.00336 (weighting factor: 1/x^2^, *r* = 0.99643) in blank brain homogenate. The LLOQ was 3.05 ng/mL in mouse plasma and mouse brain homogenate. The representative calibration curve is shown in Figure 4.

**FIGURE 5:**
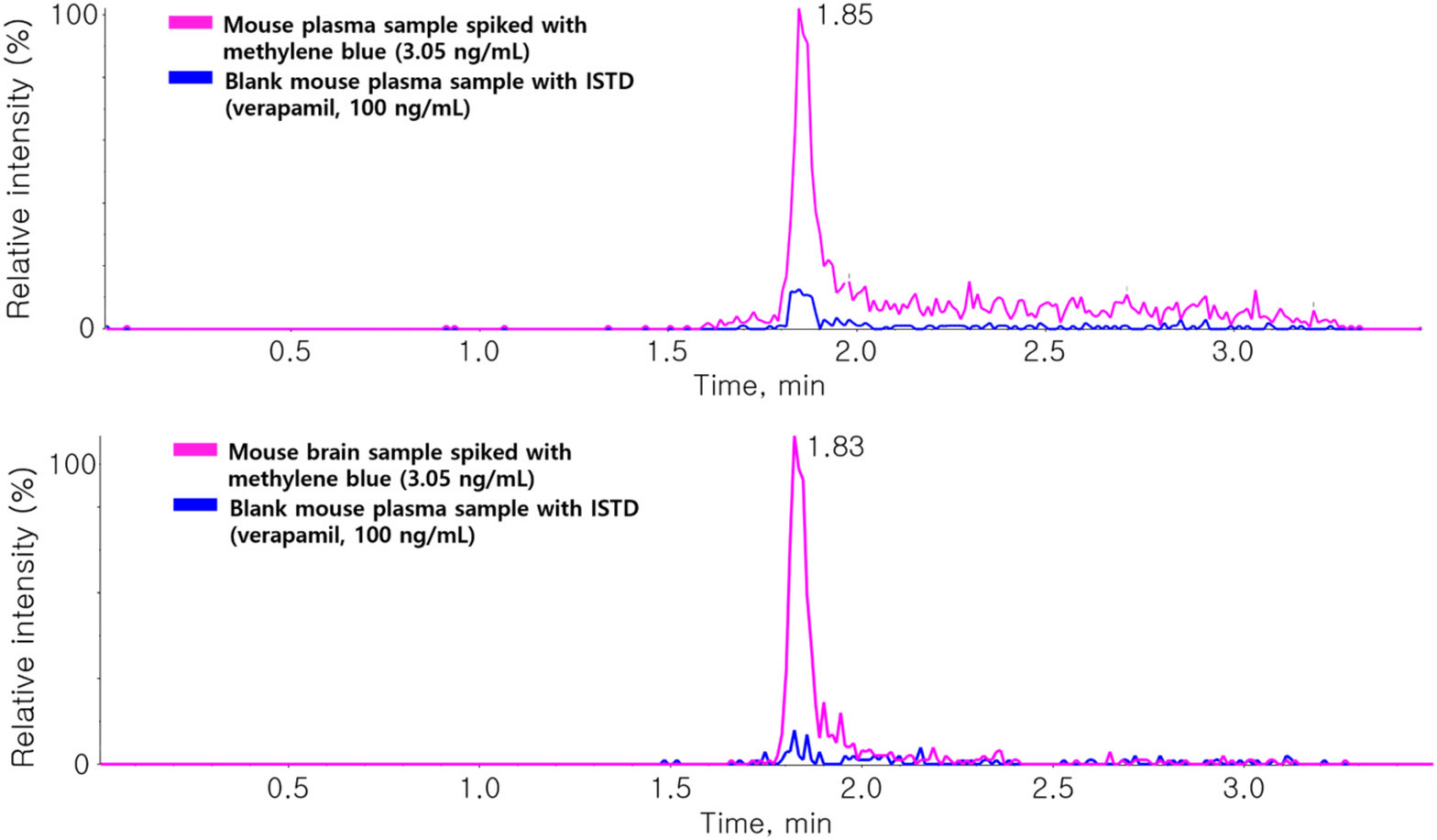
(a) Chromatogram of MB at the LLOQ (3.05 ng/mL) spiked in blank mouse plasma and (b) chromatogram of MB at the LLOQ (3.05 ng/mL) in blank mouse brain homogenate.

For selectivity and sensitivity evaluation, extracted ion chromatograms (EICs) for MB were analyzed in BI and LLOQ samples (Figure 5). Additional data, including EICs of BB, LLOQ, and the upper limit of quantification (ULOQ) samples, are provided in (Figure S1). These results confirm the absence of significant matrix effects or ISTD interference and demonstrate sufficient sensitivity for MB quantification.

#### 3.2.2 Intra‐ and Inter‐Day Accuracy and Precision Test

The intra‐ and inter‐day accuracy (RE, %) of QC samples at three concentration levels ranged from −14.17% to 13.44% in mouse plasma, and from −10.11% to 2.97% in mouse brain homogenate. The corresponding precision (RSD, %) ranged from 1.63% to 15.92% in mouse plasma and from 2.77% to 8.69% in mouse brain homogenate. These values met the in‐house acceptance criteria, confirming that this method is suitable for reproducible quantification in mouse plasma and brain homogenate (Table 2).

**TABLE 2:** The intra‐ and inter‐day accuracy and precision results in blank mouse plasma.

Intra‐day accuracy and precision test							
Run no.	Statistics	Blank mouse plasma			Blank mouse brain homogenate		
		Low QC (15.03 ng/mL)	Medium QC (165.29 ng/mL)	High QC (1818.18 ng/mL)	Low QC (15.03 ng/mL)	Medium QC (165.29 ng/mL)	High QC (1818.18 ng/mL)
Day 1 (*n* = 3)	Concentration (ng/mL)	17.05	173.46	1780.57	14.61	163.95	1872.24
	Accuracy (RE, %)	13.44	4.94	−2.07	−2.79	−0.81	2.97
	Precision (RSD, %)	1.63	2.98	3.64	8.69	5.73	5.30
Day 2 (*n* = 3)	Concentration (ng/mL)	15.93	166.48	1886.17	14.23	161.45	1769.63
	Accuracy (RE, %)	5.99	0.72	3.74	−5.32	−2.32	−2.67
	Precision (RSD, %)	7.24	7.12	6.34	2.77	3.35	5.00
Day 3 (*n* = 3)	Concentration (ng/mL)	14.44	156.53	1560.54	15.41	164.01	1634.37
	Accuracy (RE, %)	−3.93	−5.30	−14.17	2.51	−0.78	−10.11
	Precision (RSD, %)	8.23	4.09	15.92	3.78	7.29	7.96
Inter‐day accuracy and precision test							
Run no.	Statistics	Blank mouse plasma			Blank mouse brain homogenate		
		Low QC (15.03 ng/mL)	Medium QC (165.29 ng/mL)	High QC (1818.18 ng/mL)	Low QC (15.03 ng/mL)	Medium QC (165.29 ng/mL)	High QC (1818.18 ng/mL)
Day 1–Day 3 (*n* = 9)	Concentration (ng/mL)	15.81	165.49	1742.43	14.75	163.14	1758.74
	Accuracy (RE, %)	5.17	0.12	−4.17	−1.87	−1.30	−3.27
	Precision (RSD, %)	8.93	6.23	11.59	6.05	5.01	7.90

#### 3.2.3 Short‐Term (Bench‐Top), Long‐Term, and Freeze–Thaw Stability Tests

Low, medium, and high QC samples were stored under various conditions and quantified using a freshly prepared calibration curve. In mouse plasma, the accuracy (RE, %) ranged from −9.55% to 6.32%, and the precision (RSD, %) ranged from 1.26% to 15.01%. In mouse brain homogenate, the accuracy ranged from −22.32% to 2.51%, and the precision ranged from 1.20% to 6.50% (Table 3). These values met the in‐house acceptance criteria of ±25%, confirming that MB remains stable for 4 h at RT, 2 weeks at −80°C, and during three repeated freeze–thaw cycles in mouse plasma and brain homogenate.

**TABLE 3:** The short‐term (bench‐top), long‐term, and freeze–thaw stability results.

Assessment	Condition	Statistics	Blank mouse plasma			Blank mouse brain homogenate		
			Low QC (15.03 ng/mL)	Medium QC (165.29 ng/mL)	High QC (1818.18 ng/mL)	Low QC (15.03 ng/mL)	Medium QC (165.29 ng/mL)	High QC (1818.18 ng/mL)
Short‐term (bench‐top) stability (*n* = 3)	4 h, RT	Concentration (ng/mL)	15.42	153.58	1714.08	15.41	164.01	1634.37
		Accuracy (RE, %)	2.57	−7.08	−5.73	2.51	−0.78	−10.11
		Precision (RSD, %)	4.77	2.14	2.07	3.09	5.95	6.50
Long‐term stability (*n* = 3)	2 weeks, −80°C	Concentration (ng/mL)	14.75	149.51	1732.71	12.76	128.39	1498.32
		Accuracy (RE, %)	−1.86	−9.55	−4.70	−15.08	−22.32	−17.59
		Precision (RSD, %)	1.27	3.92	1.26	2.38	3.21	3.20
Freeze–thaw stability (*n* = 3)	Three cycles of freeze–thaw	Concentration (ng/mL)	15.98	165.98	1824.55	15.06	164.02	1787.96
		Accuracy (RE, %)	6.32	0.42	0.35	0.22	−0.77	−1.66
		Precision (RSD, %)	15.01	1.88	6.18	1.60	1.30	1.20

### 3.3 Pharmacokinetic Study in Male ICR Mouse

#### 3.3.1 Plasma Pharmacokinetic Study

**FIGURE 6:**
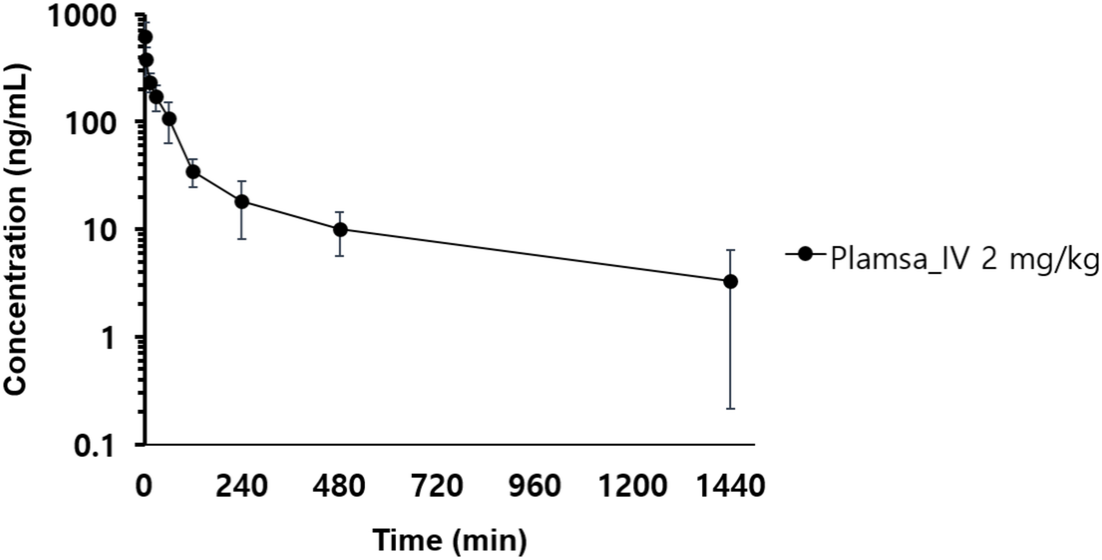
The time‐plasma concentration pharmacokinetic profile of MB following a single IV bolus injection at 2 mg/kg in male ICR mice (*n* = 4).

The developed method was applied to quantify MB plasma concentrations following a single IV bolus injection at 2 mg/kg in mice. Figure 6 presents the plasma concentration‐time profile of MB in male ICR mice, and the corresponding pharmacokinetic parameters are summarized in Table 4.

**TABLE 4:** Pharmacokinetic parameters of MB in male ICR mouse after single IV bolus injection at 2 mg/kg.

Pharmacokinetic parameters	T_max_ (min)	C_max_ (ng/mL)	AUC_last_ (min*ng/mL)	AUC_inf_ (min*ng/mL)	CL (mL/min/kg)	AUC_Extrap_ (%)	V_ss_ (mL/kg)
Mean	2.00	617.25	28,608.33	33,053.20	65.64	12.76	26,846.51
SD	0.00	223.24	10,281.61	12,096.87	18.97	9.92	27,896.02

*Note:* The time at which the C_max_ is observed (**T**_**max**_), observed maximum plasma concentration (**C**_**max**_), total drug exposure defined as area under the concentration‐time curve to the last time point (**AUC**_**last**_), total drug exposure defined as area under the concentration‐time curve extrapolated to infinity (**AUC**_**inf**_), systemic clearance (**CL**), percentage of AUC_inf_ due to extrapolation from T_last_ to infinity (**AUC**_**Extrap**_), and volume of distribution at steady state (**V**_**ss**_).

The maximum plasma concentration (C_max_) was 617.25 ng/mL, with total drug exposure, defined as the area under the concentration‐time curve extrapolated to infinity (AUC_inf_), measured at 33,053.20 min*ng/mL. The systemic clearance (CL) was 65.64 mL/min/kg, and the volume of distribution at steady state (V_ss_) was 26,846.51 mL/kg.

#### 3.3.2 Brain Distribution Study

**FIGURE 7:**
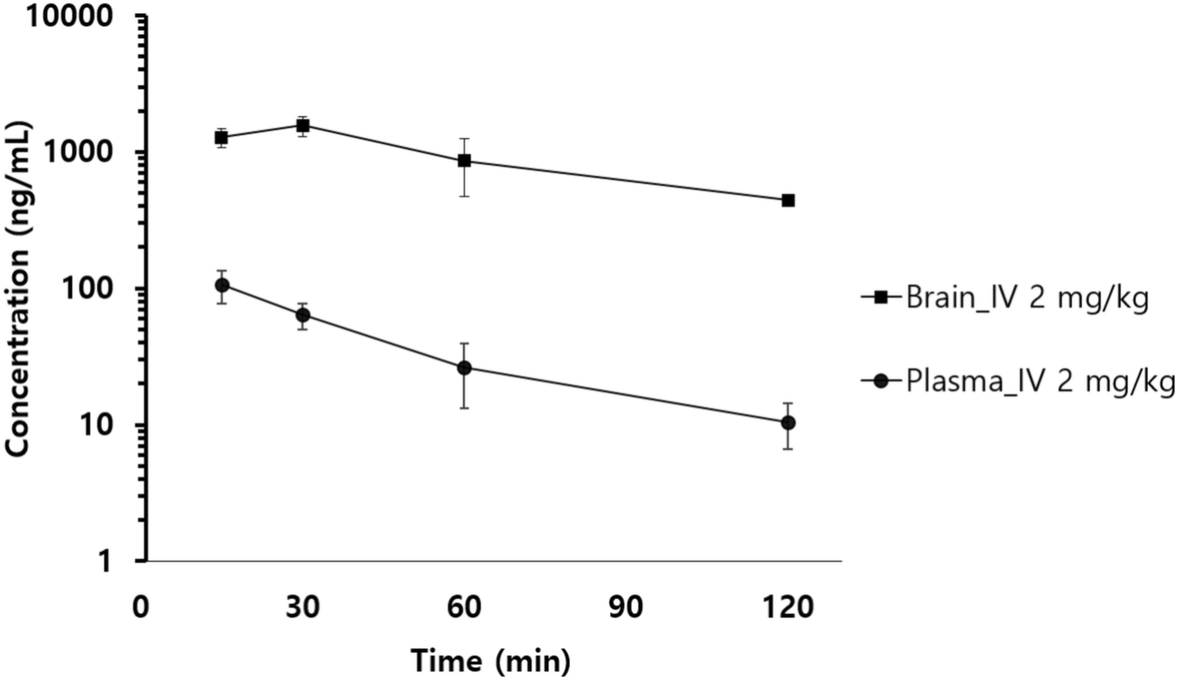
The time‐concentration profile of MB in brain and plasma following a single IV bolus injection at 2 mg/kg in male ICR mice (*n* = 3).

To characterize MB distribution in brain tissue, time‐concentration profiles in brain and plasma were obtained (Figure 7), and the *AUC*_*brain,*15–120 *min*_ and *AUC*_*plasma,*15–120 *min*_ were 106,372.28 and 4,525.58 min*ng/mL, respectively. The resulting *K*_*p,brain*_ value was 23.50 (Table 5), indicating extensive MB distribution in the brain.

**TABLE 5:** The brain‐to‐plasma partition coefficient (*K*_*p,brain*_) after single IV bolus injection at 2 mg/kg.

AUC_*brain,*15–120 *min*_ (min*ng/mL)	AUC_*plasma,*15–120 *min*_ (min*ng/mL)	*K*_*p,brain*_
106,372.28	4,525.58	23.50

## 4 Conclusions

In this study, we developed and qualified a rapid and reliable bioanalytical method using RPLC–MS for the quantification of MB in mouse plasma and brain samples. The incorporation of DFA as a mobile phase additive enabled efficient MB separation in both matrices, yielding sharp peak shapes and a short run time, making it well‐suited for pharmacokinetic studies.

Notably, the brain penetration of MB was significantly higher than expected, with a *K*_*p,brain*_ value of 23.50. Although the mechanism underlying this high brain penetration remains unclear, further studies using transporter assay systems are warranted to elucidate the pathways involved.

In conclusion, the developed method was successfully applied to plasma pharmacokinetic and brain distribution studies in male ICR mice. Beyond MB, this method is expected to be applicable to the analysis of MB analogs and other cationic basic drugs, making it a versatile tool for bioanalytical research.

## Conflicts of Interest

The authors declare no conflicts of interest.

## Supporting information


**Figure S1.** Representative chromatograms of methylene blue spiked into (a–c) mouse blank plasma and (d–f) mouse brain homogenate. Panels (a, d) show double blank samples (without analyte and internal standard), (b, e) show samples at the lower limit of quantification (LLOQ, 3.05 ng/mL), and (c, f) show samples at the upper limit of quantification (ULOQ, 2222.22 ng/mL).

## Data Availability

The datasets used and/or analyzed during the current study are available from the corresponding author on reasonable request.
