# Supplementary material for: Quantification of Methylene Blue and Evaluation of Its Pharmacokinetics in ICR Mice by Liquid Chromatography‐Quadrupole Time‐of‐Flight Mass Spectrometry Using Difluoroacetic Acid
Source: Biomed Chromatogr. 2025 Apr 20;39(6):e70080. doi: 10.1002/bmc.70080 (PMC12009784; doi:10.1002/bmc.70080)
Supplement: Supplementary file 1 — Figure S1. Representative chromatograms of methylene blue spiked into (a–c) mouse blank plasma and (d–f) mouse brain homogenate. Panels (a, d) show double blank samples (without analyte and internal standard), (b, e) show samples at the lower limit of quantification (LLOQ, 3.05 ng/mL), and (c, f) show samples at the upper limit of quantification (ULOQ, 2222.22 ng/mL). [file BMC-39-e70080-s001.docx]

| **(a)** | 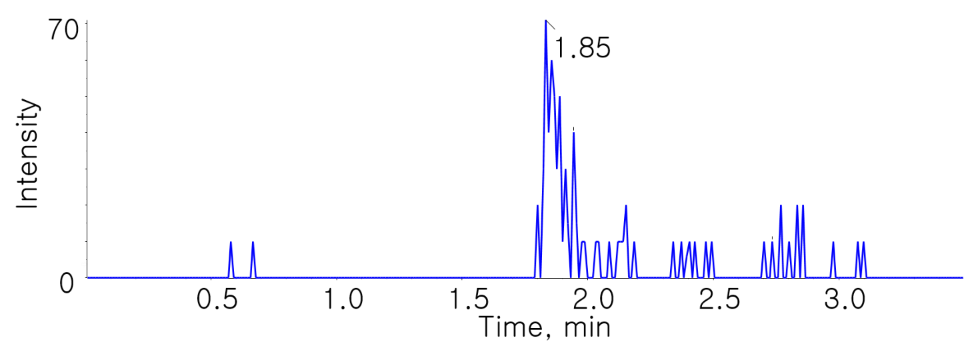 | **(d)** | 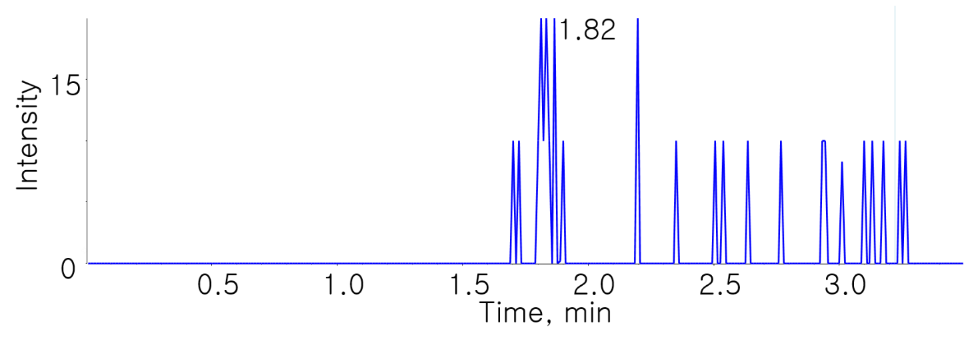 |
| --- | --- | --- | --- |
| **(b)** | 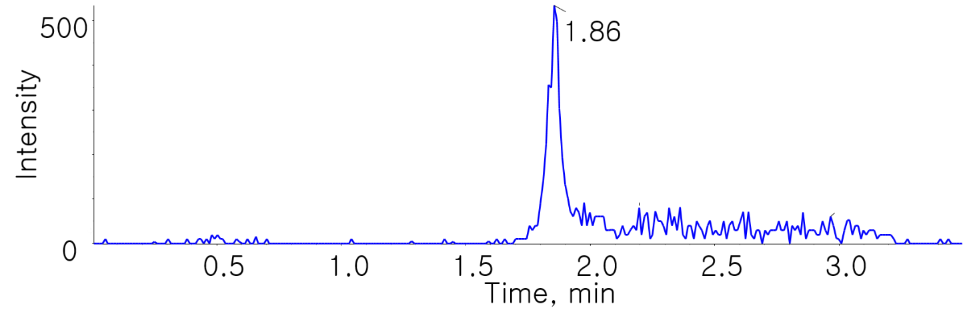 | **(e)** | 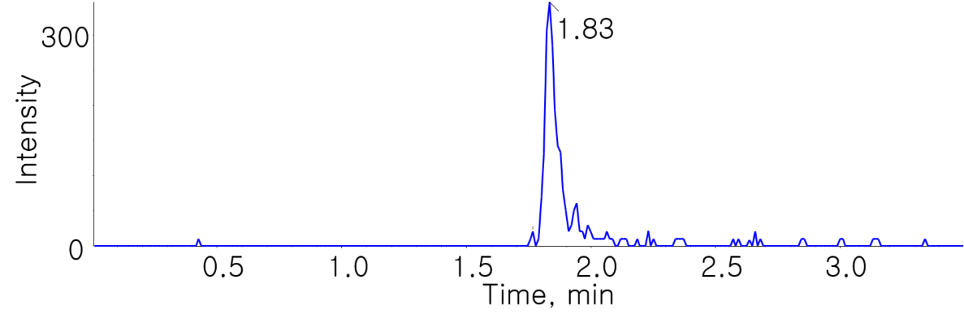 |
| **(c)** | 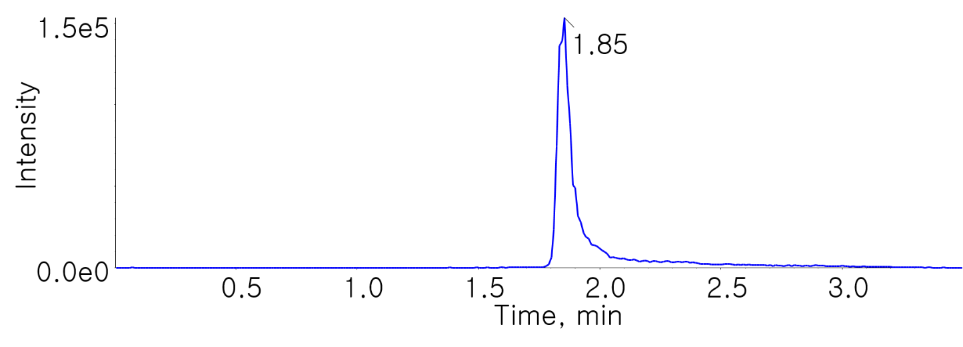 | **(f)** | 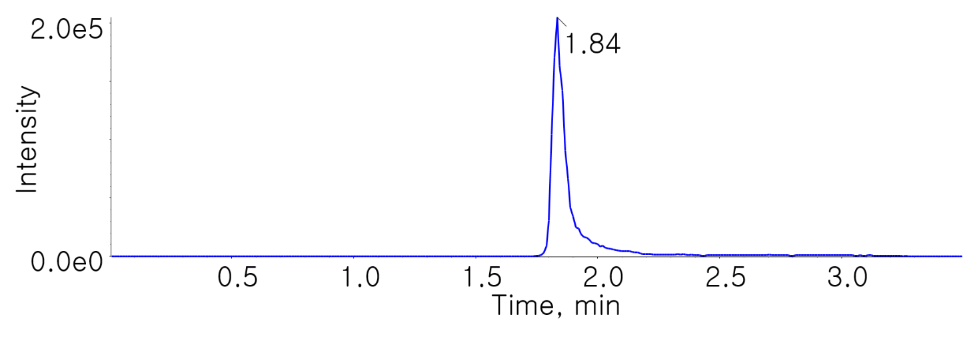 |

**Figure S-1**. Representative chromatograms of methylene blue spiked into (a–c) mouse blank plasma and (d–f) mouse brain homogenate.

Panels (a, d) show double blank samples (without analyte and internal standard), (b, e) show samples at the lower limit of quantification (LLOQ, 3.05 ng/mL), and (c, f) show samples at the upper limit of quantification (ULOQ, 2222.22 ng/mL).
